# Identifying encephalopathy in patients admitted to an intensive care unit: Going beyond structured information using natural language processing

**DOI:** 10.3389/fdgth.2023.1085602

**Published:** 2023-01-23

**Authors:** Helena Ariño, Soo Kyung Bae, Jaya Chaturvedi, Tao Wang, Angus Roberts

**Affiliations:** ^1^ Institut D’Investigacions Biomèdiques August Pi I Sunyer (IDIBAPS), Barcelona, Spain; ^2^ Institute of Psychiatry, Psychology and Neuroscience, King’s College London, London, United Kingdom; ^3^ Dept. of Integrated Medicine, Yonsei University College of Medicine, Seoul, South Korea; ^4^ Translational AI Laboratory, Yonsei University College of Medicine, Seoul, South Korea; ^5^ National Institute for Health Research, Maudsley Biomedical Research Centre, South London and Maudsley National Health Service (NHS) Foundation Trust, London, United Kingdom

**Keywords:** natural langauage processing, encephalopathy, electronic health record, ICD-9 (International classification of diseases ninth), MIMIC-III

## Abstract

**Background:**

Encephalopathy is a severe co-morbid condition in critically ill patients that includes different clinical constellation of neurological symptoms. However, even for the most recognised form, delirium, this medical condition is rarely recorded in structured fields of electronic health records precluding large and unbiased retrospective studies. We aimed to identify patients with encephalopathy using a machine learning-based approach over clinical notes in electronic health records.

**Methods:**

We used a list of ICD-9 codes and clinical concepts related to encephalopathy to define a cohort of patients from the MIMIC-III dataset. Clinical notes were annotated with MedCAT and vectorized with a bag-of-word approach or word embedding using clinical concepts normalised to standard nomenclatures as features. Machine learning algorithms (support vector machines and random forest) trained with clinical notes from patients who had a diagnosis of encephalopathy (defined by ICD-9 codes) were used to classify patients with clinical concepts related to encephalopathy in their clinical notes but without any ICD-9 relevant code. A random selection of 50 patients were reviewed by a clinical expert for model validation.

**Results:**

Among 46,520 different patients, 7.5% had encephalopathy related ICD-9 codes in all their admissions (group 1, definite encephalopathy), 45% clinical concepts related to encephalopathy only in their clinical notes (group 2, possible encephalopathy) and 38% did not have encephalopathy related concepts neither in structured nor in clinical notes (group 3, non-encephalopathy). Length of stay, mortality rate or number of co-morbid conditions were higher in groups 1 and 2 compared to group 3. The best model to classify patients from group 2 as patients with encephalopathy (SVM using embeddings) had F1 of 85% and predicted 31% patients from group 2 as having encephalopathy with a probability >90%. Validation on new cases found a precision ranging from 92% to 98% depending on the criteria considered.

**Conclusions:**

Natural language processing techniques can leverage relevant clinical information that might help to identify patients with under-recognised clinical disorders such as encephalopathy. In the MIMIC dataset, this approach identifies with high probability thousands of patients that did not have a formal diagnosis in the structured information of the EHR.

## Introduction

Encephalopathy is an umbrella term that comprises a constellation of neurocognitive conditions ranging from an acute confusional state (*delirium*) to a decrease of consciousness or more subtle acute changes in personality (1). It can be the result of a primary brain disorder such as inflammation (*encephalitis*) and is also a frequent complication of severe toxic-metabolic disorders such as sepsis. It is particularly prevalent among patients needing intensive care, aggravating the outcome when it occurs as comorbidity. Delirium prevalence varies considerably by patient group and setting. The prevalence of delirium is relatively high in intensive care unit (ICU) patients; 32% in ventilated and non-ventilated intensive care unit (ICU) patients and 50%–70% in mechanically ventilated patients (2). Recognizing the disorder and understanding its pathophysiology has important clinical implications since acute encephalopathy is associated with a higher risk of death, longer hospital stay, and disability, in particular dementia (2). Nevertheless, diagnosis of encephalopathy in the clinical setting is challenging for several reasons, greatly because there are no formal criteria to establish a diagnosis with encephalopathy and because the phenotype depends on the underlying physiopathology and severity. This poses an important limitation when trying to leverage knowledge in retrospective studies. In fact, previous studies have been focused on detecting and predicting either delirium (3, 4), a syndrome with well-characterized clinical criteria (5) and specific screening tools (6), or coma, the most severe form of encephalopathy. However, persistent cognitive impairment at 12 months after intensive care unit (ICU) discharge occurs also in patients that have not received a diagnosis with delirium (7). Neglecting intermediate forms of encephalopathy may introduce bias in predictive studies and miss a complete perspective of the implications of encephalopathy.

A further limitation addressing studies with encephalopathy is that diagnostic codes for encephalopathy in the electronic health record (EHR) are not reliable, and even the most recognizable syndromic presentation (delirium) is underrepresented in the structured diagnostic information of patients as International Classification of Diseases (ICD) (8) codes (9–11). As an alternative, some authors have used the Confusion Assessment Method for ICU (CAM-ICU), the most widespread scale to screen for delirium (12).

To address the issues of misclassifications, imprecision and omissions in diagnostic codes, recent studies have exploited machine learning methodologies to detect risk of encephalopathy and delirium (ED) based on patient information recorded in electronic health records (EHRs) (10, 11, 13–16). For example, Corradi et al. used a Random Forest model based on demographic data, comorbidities, medications, procedures, and physiological measures to predict ED (15), and Racine et al. showed that machine learning methods can be used to identify patients at high risk of developing delirium after surgery (16). These methods have largely focused on modeling structured EHR data, i.e., entries with a pre-defined format such as patient demographics and lab tests. However, EHRs contain rich information describing all aspects of the health and care of hospitalized patients in the form of unstructured free text, such as clinical notes. It is likely that disclosing this information may enrich predictors or classifiers, because the semiology (the group of signs and symptoms that characterize ED), or clinical scales regarding ED are generally only recorded in clinical notes when clinicians make assessments.

To unlock the potentials of clinical text and address the aforementioned limitations of using structured clinical information, Natural language processing (NLP), a set of methods and techniques for the computational processing of text, and machine learning have fast evolved in recent years so the aforementioned limitations of using structured clinical information might be partially overcome. In the field of ED, NLP has demonstrated that neither ICD codes or specific delirium clinical scales are sensitive enough to capture all the phenotypic range of encephalopathy. In particular, behavioural disturbances captured by NLP encloses more patients at risk of receiving antipsychotic medications or having higher morbimortality rates in the ICU than the group of patients defined by the CAM-ICU (17). Recent approaches have tried to incorporate keywords related to the delirium semiology into machine learning classifiers to label patients with delirium (10, 18). For example, Coombes et al. used 8 words from the clinical notes text (“AMS”, “mental status”, “deliri”, “hallucin”, “confus”, “reorient”, “disorient”, “encephalopathy”) among other changes in clinical actions manually selected (10). However, predefined keywords can hinder the discovery of new insights and cannot be seamlessly generalized to other datasets due to potentially different language use in other settings. Also, keyword matching cannot take contextual meaning of a term into account and hence can return irrelevant information such as misspelling and ambiguous language in EHR (e.g., “AIDS” can refer to “Acquired Immune Deficiency Syndrome” but also hearing aids).We aim to investigate in this study the potential of state-of-the-art NLP, to identify patients with encephalopathy in EHRs of patients admitted to intensive care units. We hypothesize that considering all clinical concepts contained in the free text (not only those related to the delirium semiology) and a more complex language model representation to capture semantic similarities (word embeddings instead of string matching) (19, 20) could yield better results to detect patients with different phenotypes of encephalopathy besides delirium.

## Materials and methods

**Figure 1:**
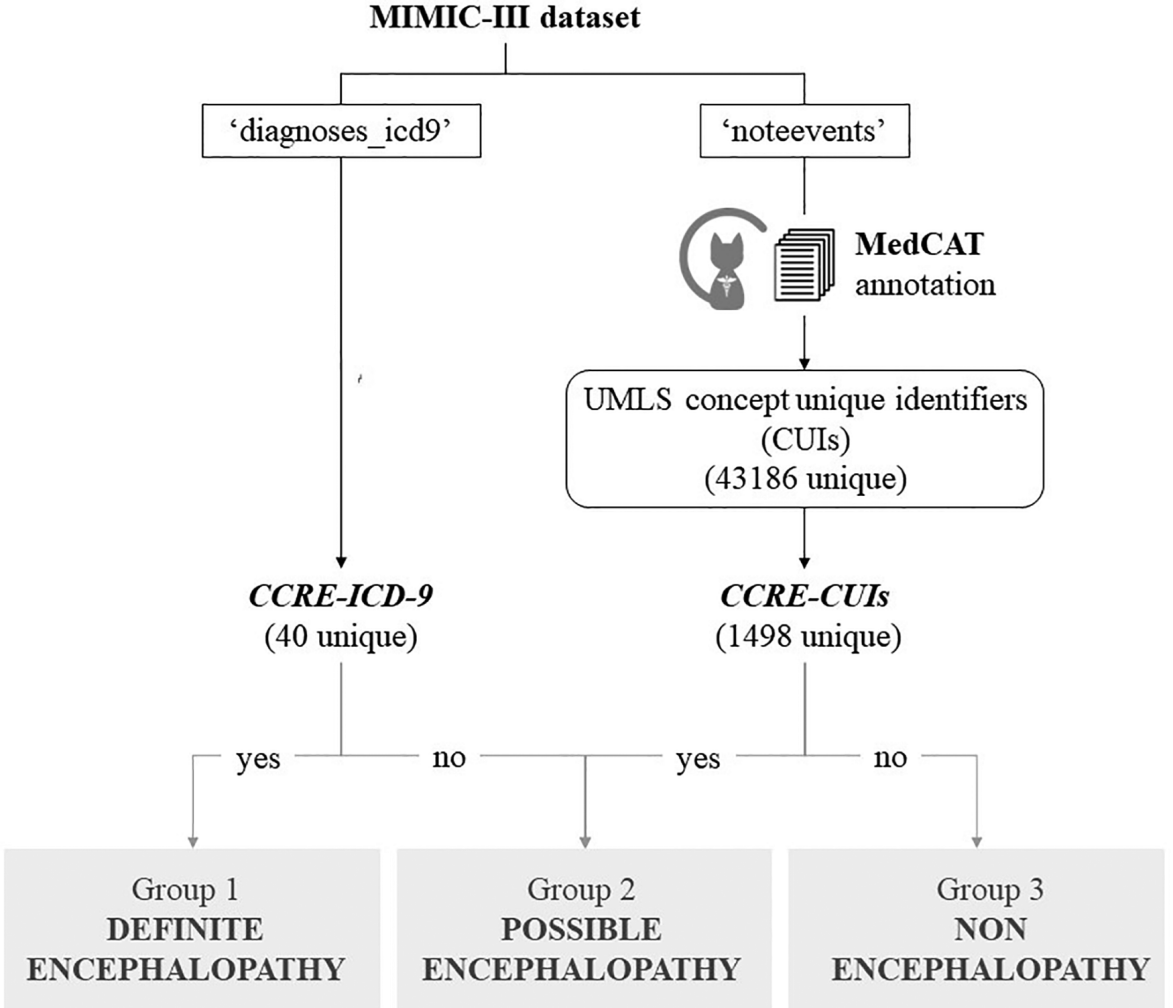
Cohort definition. Flowchart representing the classification of patients from MIMIC-III dataset, based on their encephalopathy status. Groups 1 + 3 were used as positive/negative label to train the model in a binary classification task. The best model was applied to unseen cases with possible encephalopathy.

**Figure 2:**
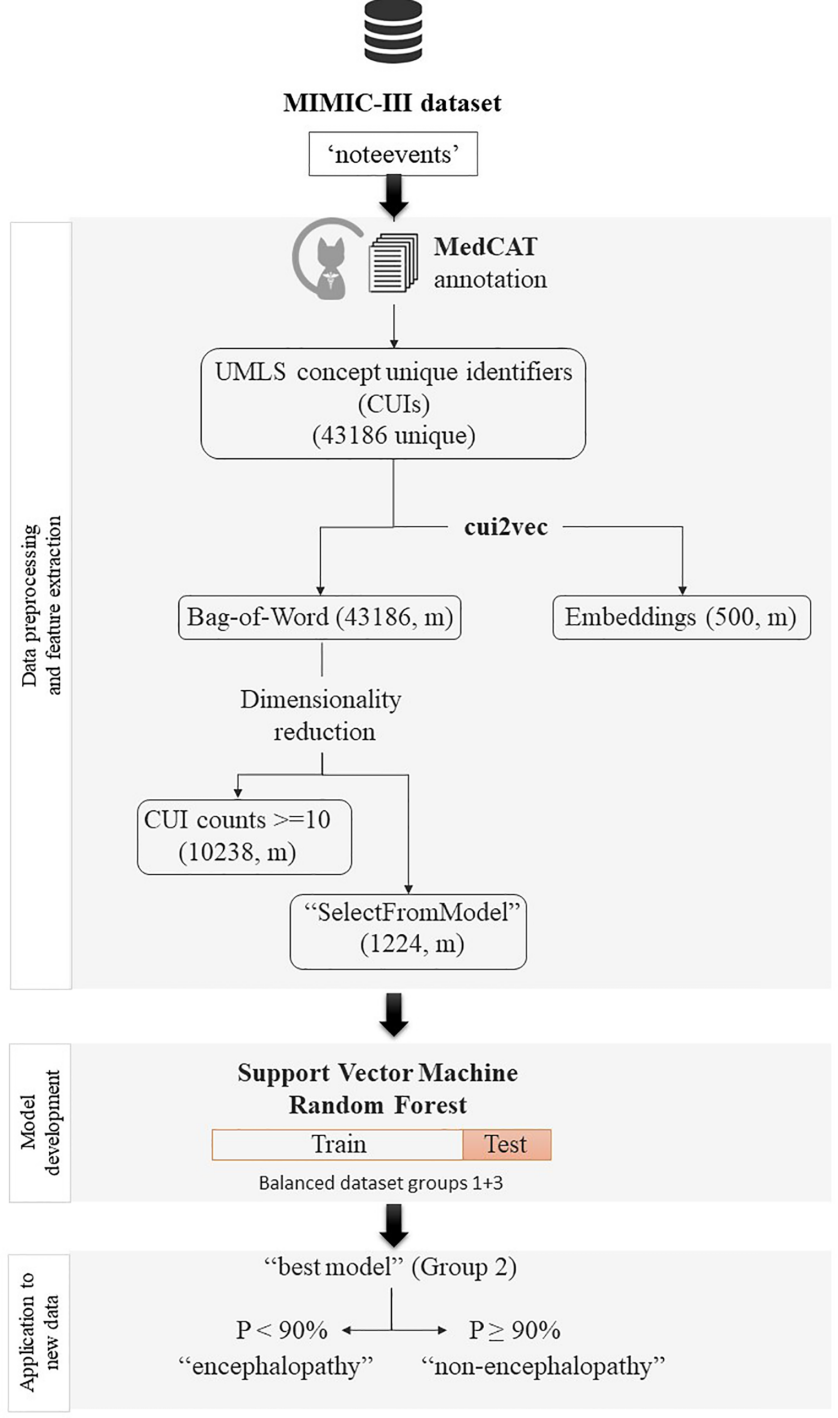
Flowchart of pre-processing, cohort definition and classification. Graph that sumarises the flowchart of processing of the data, from the inclusion criteria to the final binary classification task to identify encephalopathy among patients without a formal diagnosis.

Figures 1, 2 summarise the flowchart of methods applied.

### Patients and source of data

We analysed the MIMIC-III dataset (21), a freely accessible critical care database of 46,520 patients admitted between 2001 and 2012 in a single centre in the United States. From the available data, we used both the unstructured text notes in the “noteevents” table and the structured diagnosis recorded as ICD-9 codes from the “diagnoses_icd9” table from all patients with at least 1 clinical note in the “noteevents” table without exclusion criteria.

Encephalopathy was defined based on ICD diagnostic codes (see next paragraph). For expert validation, “encephalopathy” was considered when a change in mental state was reported, including progressive cognitive dysfunction of more than 1 cognitive domain, personality changes, inattention, or consciousness impairment (from somnolence to coma).

### List of clinical concepts related to encephalopathy (CCRE)

From an initial list of signs, symptoms, and ICD-9 diagnostic codes indicative of encephalopathy selected by a clinical expert, these concepts were mapped into a standardized vocabulary, Unified Medical Language System (UMLS) (22)), and further expanded to include child concepts of the terms from the initial list using Clinical Knowledge Graph (23). This process (Figure 1 and Supplementary Material S1) generated 2 final lists of clinical concepts related to encephalopathy that were used to classify MIMIC's patients, one containing relevant ICD-9 codes (CCRE-ICD-9, Table 1), and a second one containing 1,498 UMLS concept unique identifiers (CUIs) for encephalopathy (CCRE-CUIs).

**Table 1:** List of CCRE-ICD-9.

No.	CUIs	Name	ICD9
1	C0859643	Senile dementia with delusional or depressive features	290.2
2	C0154315	Senile dementia with delirium	290.3
3	C0154319	Other specified senile psychotic conditions	290.8
4	C1457889	Unspecified senile psychotic condition	290.9
5	C0001957	Alcohol withdrawal delirium	291.0
6	C0152128	Drug withdrawal	292.0
7	C0152129	Pathological drug intoxication	292.2
8	C1456296	Delirium due to conditions classified elsewhere	293.0
9	C0154333	Subacute delirium	293.1
10	C1456302	Unspecified transient mental disorder in conditions classified elsewhere	293.9
11	C0085584	Encephalopathy, not elsewhere classified	348.3
12	C0154309	Presenile dementia with delirium	290.11
13	C0236651	Vascular dementia, with delirium	290.41
14	C0236652	Vascular dementia, with delusions	290.42
15	C1456286	Drug-induced psychotic disorder with delusions	292.11
16	C1456732	Drug-induced psychotic disorder with hallucinations	292.12
17	C0154326	Drug-induced delirium	292.81
18	C1456288	Drug-induced persisting dementia	292.82
19	C1456297	Psychotic disorder with delusions in conditions classified elsewhere	293.81
20	C0029226	Psychotic disorder with hallucinations in conditions classified elsewhere	293.82
21	C1456298	Mood disorder in conditions classified elsewhere	293.83
22	C1456299	Anxiety disorder in conditions classified elsewhere	293.84
23	C0154334	Other specified transient mental disorders due to conditions classified elsewhere, other	293.89
24	C0006112	Metabolic encephalopathy	348.31
25	C1260408	Other encephalopathy	348.39
26	C0149504	Toxic encephalopathy	349.82
27	C0221539	Transient alteration of awareness	780.02
28	C0221540	Other alteration of consciousness	780.09
29	C0278061	Altered mental status	780.97
30	C0018524	Hallucinations	780.1
31	C0009421	Coma	780.01
32	C0752304	Hypoxic-ischemic encephalopathy (HIE)	768.7
33	C0752304	Hypoxic-ischemic encephalopathy, unspecified	768.70
34	C0019151	Hepatic encephalopathy	572.2
35	C0302369	Alcohol-induced psychotic disorder with hallucinations	291.3
36	C0236656	Alcohol-induced persisting dementia	291.2
37	C0085584	Encephalopathy, unspecified	348.30
38	C0151620	Hypertensive encephalopathy	437.2
39	C2712360	Severe hypoxic-ischemic encephalopathy	768.73
40	C1269750	Senile dementia with delusional features	290.20

### Feature extraction from clinical notes

All clinical text notes in the MIMIC-III database (“noteevents”) were automatically annotated by MedCAT, a NLP tool that extends sciSpaCy for Named Entity Recognition of clinical concepts, linking these to the standard nomenclatures SNOMED Clinical Terms and UMLS (24). MedCAT also generates “status” labels for each annotation to ensure that annotated concepts are contextually relevant. For example, extracted concepts may need to be ignored if they appeared in the past or are negated. We used a public MedCAT model that was pre-trained based on all text data in the MIMIC-III database using a vocabulary of medical concepts defined in UMLS. The MedCAT has been validated using real-world EHR data from 3 large London hospitals (including both acute and mental health hospitals) and has shown consistently good performance across hospitals, datasets and medical concept types and it achieved precision rates (F1) above 0.90 for extracting 21 common physical comorbidities in an independent study (25). Concepts that were mentioned in a clinical notes, identified as CUIs by MedCAT, were used as features of the note. Based on these notes’ features, we represent a patient profile through two widely used approaches. The first one is the Bag-of-Words presentation, in which the frequency of each CUI in all clinical notes of a patient is used as a feature of the patient's profile. The second approach is a word embedding presentation, in which each CUI in a patient's clinical notes is mapped to an embedding vector and vectors of all CUIs are aggregated by an average operation. To reduce dimensionality of the resulting sparse matrix, we performed feature selection based on a minimum threshold of occurrence in the whole corpus (counts ≥ 10) or through an off-the-shelf meta-transformer for selecting features based on importance weight with the “mean” as threshold for selection (“SelectFromModel” method of Scikit Learn). Here, we used cui2vec, an embedding model pre-trained on a large collection of multimodal medical data (26).

### Analysis

Admissions were aggregated by unique patients and patients were classified into 4 groups based on findings of CCRE across their admissions: Group 1) *definite encephalopathy cohort*: those with a formal diagnosis of encephalopathy defined as CCRE-ICD-9 (Table 1) in all episodes, Group 2) *possible encephalopathy cohort*: patients with CCRE-CUIs in their clinical notes but without a formal diagnosis in their structured fields (no CCRE-ICD-9), Group 3) *non-encephalopathy cohort:* patients with no relevant concepts neither in the structured nor the unstructured information, Group 4) *mixed cohort*: patients having more than 1 episode with discordant criteria between episodes who cannot be classified in any of the above. This latest group was expected to be similar to group 1 in terms of demographic features, however downstream supervised machine learning tasks were performed using admissions (not patients) as independent instances and this group of patients with mixed type of admissions were excluded.

Non-parametric hypothesis tests (Mann–Whitney *U* test and Kruskal–Wallis test) were used to evaluate differences in proportions and median values for descriptive analysis of the different groups of patients. Machine learning classifiers [support vector machines with different kernel options (linear, RBF, and sigmoid) and random forest algorithms with different feature selection strategies] to predict patients with a high probability of having encephalopathy. For this binary classification task at the admission level a formal diagnosis of encephalopathy during an admission (meaning having at least 1 CCRE-ICD-9, group 1) was considered the gold-standard for positive class, while those admissions from patients that were never diagnosed with encephalopathy or never had any related clinical concept in their notes (group 3) were label as negative cases. Given the predominance of negative cases (6:1), we created a balanced dataset (1:1) after a random selection of negative cases to develop different classifiers by cross-validation (train-test sets: 80%–20%).The best predictive model was selected based on its F1 measure in the test set and applied to classify unseen and unlabeled cases over a random selection of potential admissions with encephalopathy (group 2, those patients with CCRE in clinical texts but not a formal diagnosis). We classified patients with encephalopathy when the probability of the model output was 90% or higher and validation of this output was done by a clinical expert (neurologist) in a random sample of 50 cases.

Python 3 was used for the analysis. Some of the partial results of the pipeline can be reached in a public repository (https://github.com/skwgbobf/Publication).

## Results

### MIMIC cohort

There were 46,520 different patients admitted to ICU with 58,976 different admissions. CCRE-ICD-9 codes were retrieved in 16,693 (28.3%) admissions and 7,927 (17%) different patients from structured fields. Among all admissions, CCRE-CUIs were found in 28,620 (61.5%) patients' clinical notes. Table 2 summarizes the different cohorts of patients based on the combination of these findings and some basic epidemiological data. In general terms, patients from Groups 1 and 2 were older, had longer stays, a wider list of different diagnoses during the episode, and higher mortality rates compared to patients without encephalopathy (Group 3). This suggests on the one hand that patients with encephalopathy have higher clinical complexity and severity, and on the other hand, that patients with only unstructured information regarding encephalopathy had similar features to the cohort of patients with CCRE-structured information.

**Table 2:** Classification of MIMIC patients based on their structured and unstructured information.

	Group 1 (definite E)	Group 2 (possible E)	Group 3 (non-E)	Group 4 (both type of episodes)	*p*-value among the 4 groups (*χ*^2^-test or Kruskal–Wallis)	*p*-value between groups 1&2 (*χ*^2^-test or *U* Mann–Whitney)
Patients, *n* (% over total cohort)	3,506 (7.5%)	20,906 (44.9%)	17,687 (38.0%)	4,421 (9.5%)	n/a	n/a
Age, median (IQR)	66.23 (52.3–79.2)	66.05 (50.0–78.9)	49.94 (0–68.2)	64.54 (52.5–76.1)	<0.001	<0.001
Gender, F (%)	1,481 (42.2%)	9,281 (44.4%)	7,694 (43.5%)	1,943 (43.9%)	0.069	0.018
Ethnicity, white (%)	2,653 (73.1%)	14,786 (70.6%)	11,734 (66.2%)	3,350 (73.2%)	<0.001	0.011
Mortality, *n* (%)	1,528 (43.6%)	8,889 (42.5%)	3,016 (17.1%)	2,326 (52.6%)	<0.001	0.246
Length of stay, median days (IQR)	12 (7–21)	9 (5–16)	6 (4–10)	8 (5–15)	<0.001	<0.001
Re-admission, *n* (%)	163 (4.6%)	1,975 (9.4%)	978 (5.5%)	4,421 (100%)	<0.001	1.89
Diagnosis, median (IQR) number of different ICD9/admission	9 (7–14)	16 (11–21)	7 (4–10)	12.6 (9–16.5)	<0.001	<0.001

### Text features

To use clinical notes as input in classifiers for encephalopathy, we used the output of the default MedCAT annotation. A validation exercise annotating 50 documents demonstrated a good performance of the MedCAT model in identifying medical concepts from clinical text (Supplemental Material). We obtained a total number of 43,186 different CUIs (94 CCRE-CUIs, 0.2%). Both groups of patients had a similar number of clinical notes, that were written in its majority by nursing staff. However, the structured cohort had a higher number of annotated clinical concepts and a slightly higher frequency of physician's notes compared to Group 2.

Regarding CCRE-CUIs, a short list encompassed the majority of counts. The top ten in each group accounted over 90% of all the counts, and the list of the most prevalent CUIs was very similar between the 2 groups, as 8 of the 10 CUIs were shared by both groups. (Table 3).

**Table 3:** Comparison of text features between structured and unstructured cohorts.

	Group 1 (structured)	Group 2 (only unstructured)	*p-*value between groups 1&2 (*χ*^2^-test or *U* Mann–Whitney)
Median number of clinical notes/patient, IQR	24 (12–48)	22 (12–46)	0.008
Median number of CUIs/note, IQR	67 (37–134)	49 (20–86)	<0.001
Distribution of type of clinical notes			<0.001
Physician, *n* (%)	21,171 (13.8%)	67,632 (6.8%)	
Nursing, *n* (%)	61,534 (40.2%)	597,211 (60%)	
Radiology, *n* (%)	42,969 (28.1%)	194,259 (19.5%)	
ECG, *n* (%)	11,248 (7.3%)	62,994 (6.3%)	
Others, *n* (%)	16,134 (10.5%)	72,637 (7.3%)	
Top 10 CCRE-CUIs (% of total CCRE-CUIs)	1. C0085631_[Agitation]	1. C0085631_[Agitation]	
	2. C0278061_[Abnormal mental state]	2. C0009676_[Confusion]	
	3. C0011206_[Delirium]	3. C0023380_[Lethargy]	
	4. C0009676_[Confusion]	4. C0278061_[Abnormal mental state]	
	5. C0023380_[Lethargy]	5. C0011206_[Delirium]	
	6. C0237284_[unresponsive behavior]	6. C0237284_[unresponsive behavior]	
	7. C0019151_[Hepatic Encephalopathy]	7. C0039070_[Syncope]	
	8. C0001957_[Alcohol Withdrawal Delirium]	8. C0233407_[Disorientation]	
	9. C0236663_[Alcohol withdrawal syndrome]	9. C0001957_[Alcohol Withdrawal Delirium]	
	10. C0085584_[Encephalopathies]	10. C0236663_[Alcohol withdrawal syndrome]	
	(92.2%)	(92.8%)	

### Classifying cases with a high probability of encephalopathy based on the unstructured information

We used supervised machine learning to build a binary classifier to determine whether a patient had encephalopathy. The classifiers were trained on a cohort of 7,308 admissions (6,922 unique patients from groups 1 and 3) using CUIs (not only CCRE) of clinical notes as features either in a Bag-of-Words or Embeddings representation.

We applied different feature selection strategies to reduce the high dimensionality and sparsity of the resulting matrix in the Bag-of-Words representation and kept the 500-dimensional embeddings resulting from the cui2vec transformation. Among all models explored, we found that a Support Vector Machine with Linear or RBF Kernel with cui2vec features achieved the highest F1 and precision scores in the 5-fold cross-validation (highlighted in bold in Table 4), and called the “best model” from now on.

**Table 4:** Models to classify encephalopathy.

Model	Feature selection	Number of features	F1 (5-fold cross validation)	Precision for class 1
Linear SVM	≥10 counts	10,238	82%	83%
Linear SVM	≥10 counts, “SelectFromModel”	1,224	78%	80%
**Linear SVM**	**cui2vec**	**500**	**85%**	**84%**
**RBF SVM**	**cui2vec**	**500**	**85%**	**84%**
Sigmoid SVM	cui2vec	500	83%	77%
Random Forest	≥10 counts	10,238	82%	81%
Random Forest	cui2vec	500	83%	81%

The application of the best model to a random sample of 7,155 admissions (5,710 unique patients) from Group 2 predicted 4,735 (66.2%) admissions (3,871 unique patients) with a probability of >50%, and 1,980 (27.7%) admissions (1,771 unique patients) with a probability of >90% among them. The random sample rather than the entire Group 2 was used due to the limit of computational resources in processing the entire dataset. These results lead to an estimation of 31.1% (95% CI: 29.8%–32.2%) of encephalopathy prevalence among patients admitted in the ICU without a formal diagnosis.

Validation of this model was performed by a clinical expert in a random sample of 50 patients selected among those without relevant structured information (group 2) and a predicted probability above 90%. This validation found that 49/50 had symptoms suggestive of encephalopathy (precision of 98%). However, 3 of them were very transient symptoms (“confused”, “agitated”) in the context of pain or decreased level of consciousness (“lethargic”) in the context of pharmacological sedation which probably represent physiological reactions to drugs or nociceptive stimuli rather than a pathological mental state, so only 46 patients would be considered encephalopathic by a clinical expert (precision of 92%). Among these 50 patients, only 21 (42%) had an acute brain or intracranial process that could justify the abnormal mental state.

## Discussion

The results of this study suggest that: (1) encephalopathy is poorly coded in the structured diagnosis even in a high-risk context such intensive care units; (2) that word embeddings pretrained over clinical concepts (Clinical Concept Embeddings) are more accurate to capture the semantics related to encephalopathy than a Bag-of-Words representation, and that (3) the off-the-shelf library MedCAT for Named Entity Recognition and linkage can be used to annotate concepts in clinical texts providing meaningful results even without fine tuning.

We annotated (and normalized to a standard ontology) clinical concepts from over 40 thousand patients admitted in the ICU, with a median number of 23 clinical notes per patient and around 50 clinical concepts per note, with a minimum effort. A pre-defined list of keywords only allows to select a limited amount of data by hypothesis driven feature selection. This would preclude necessary steps to escalate this methodology such as merging sources of data with different feature format (different languages, for example) in a federate learning framework or to validate this model in external datasets. Although the accuracy of the raw MedCAT model was over 90% in different meta-annotation metrics (see Supplementary Material), it is likely that a fine-tune step could yield better results, especially in the NER + L task.

Among the models explored, those using clinical word embeddings obtained the best performance. We used pretrained embeddings, learned using an extremely large collection of multimodal medical data, which can vectorize 108,477 clinical concepts from its UMLS identifier (CUI) and permit a quick feature extraction for medical concepts, independent of the language. This ready-to-use tool poses an important advantage in contexts of scarce labeled healthcare data unable to provide enough contextual information to encode semantics in new trained clinical embeddings (26). In combination with MedCAT, it might overcome the barriers to share data in different languages. Moreover, it offers at the same time a dimensionality reduction solution. The result is an output of a 500-dimensional embedding per concept, in contrast to higher dimensional vectors resulting from one-hot encoding (in our case, 43,186 CUIs). Representing documents as aggregated vectors keeps this low-dimensionality in favour of more efficient models, at the expense of explainability. We could not explore which clinical concepts are more important to predict the label encephalopathy.

However, they served to correctly classify patients with encephalopathy. We first screen those clear negative patients without any suspicion of encephalopathy given the absence of any relevant ICD code or any relevant clinical concept (CCRE-CUIs) in clinical notes. After defining cohorts with definite, possible and absence of encephalopathy, we trained classic machine learning algorithms and could classify patients with high probability of having encephalopathy among those without a formal diagnosis in their structured fields (those patients with possible encephalitis). Previous studies used biological validation to evaluate the reclassification of patients as delirium, by clinically meaningful outcomes such as mortality) (10). Besides that, here we use a domain-expert validation to evaluate the performance of the model over new data. Although the best model had a precision of 84% in the test set, selecting a high threshold (probability of the prediction of 90% or more) enhanced the precision in the application of the model to unseen data. We reclassify around 30% of new patients without a previous formal diagnosis of encephalopathy, meaning thousands of patients of the MIMIC-III cohort. This confirms that ICD codes, that were designed for billing purposes, are not a good criteria for cohort definition used in retrospective research studies (27, 28). Moreover, our study suggests that encephalopathy is associated with higher mortality either the patient receives a formal diagnosis or not, so this disorder shouldn't be overlooked. For this model to further develop into a real-time computer-aided alarm system, we should explore the generalizability of these results in new clinical settings. We don't know how representative the clinical notes from patients in this dataset. Other authors have used the MIMIC-III dataset to validate models developed in a different institution in the United States (13), but it is likely that higher discrepancies with critically ill patients in other countries beyond the United States with different assistance protocols exist. In addition, the performance of different NER + L methods can vary on different datasets.

To conclude, we show here how state-of-the-art NLP techniques can help to identify patients with under-reported clinical disorders such as encephalopathy. In the MIMIC dataset, this approach identifies with high probability thousands of patients that did not have a formal diagnosis in the structured information of the EHR.

## Data Availability

The original contributions presented in the study are included in the article/**Supplementary Material**, further inquiries can be directed to the corresponding author.

## References

[1] Slooter AJC Otte WM Devlin JW Arora RC Bleck TP Claassen J Updated nomenclature of delirium and acute encephalopathy: statement of ten societies. Intensive Care Med. (2020) 46:1020–2. 10.1007/s00134-019-05907-4 32055887 PMC7210231

[2] Wilson JE Mart MF Cunningham C Shehabi Y Girard TD MacLullich AMJ Delirium. Nat Rev Dis Primers. (2020) 6(1):90. 10.1038/s41572-020-00223-4 33184265 PMC9012267

[3] van den Boogaard M Pickkers P Slooter AJC Kuiper MA Spronk PE van der Voort PHJ Development and validation of PRE-DELIRIC (PREdiction of DELIRium in ICu patients) delirium prediction model for intensive care patients: observational multicentre study. BMJ. (2012) 344:e420. 10.1136/bmj.e420 22323509 PMC3276486

[4] Wong A Young AT Liang AS Gonzales R Douglas VC Hadley D. Development and validation of an electronic health record-based machine learning model to estimate delirium risk in newly hospitalized patients without known cognitive impairment. JAMA Netw Open. (2018) 1(4):e181018. 10.1001/jamanetworkopen.2018.1018 30646095 PMC6324291

[5] American Psychiatric Association. Diagnostic and Statistical Manual of Mental Disorders. American Psychiatric Association Publishing (2022). Available at: https://psychiatryonline.org/doi/book/10.1176/appi.books.9780890425787.

[6] Ely EW Inouye SK Bernard GR Gordon S Francis J May L Delirium in mechanically ventilated patients: validity and reliability of the confusion assessment method for the intensive care unit (CAM-ICU). JAMA. (2001) 286(21):2703–10. 10.1001/jama.286.21.2703 11730446

[7] Bulic D Bennett M Georgousopoulou EN Shehabi Y Pham T Looi JCL Cognitive and psychosocial outcomes of mechanically ventilated intensive care patients with and without delirium. Ann Intensive Care. (2020) 10(1):104. 10.1186/s13613-020-00723-2 32748298 PMC7399009

[8] World Health Organization. ICD-9-CM: International classification of diseases, 9th revision, clinical modification (1996). Available at: https://icd.who.int/.

[9] Horsky J Drucker EA Ramelson HZ. Accuracy and completeness of clinical coding using ICD-10 for ambulatory visits. AMIA Annu Symp Proc. (2017) 2017:912–20. PMID: 29854158 29854158 PMC5977598

[10] Coombes CE Coombes KR Fareed N. A novel model to label delirium in an intensive care unit from clinician actions. BMC Med Inform Decis Mak. (2021) 21(1):97. 10.1186/s12911-021-01461-6 33750375 PMC7941123

[11] Kim DH Lee J Kim CA Huybrechts KF Bateman BT Patorno E Evaluation of algorithms to identify delirium in administrative claims and drug utilization database. Pharmacoepidemiol Drug Saf. (2017) 26(8):945–53. 10.1002/pds.4226 28485014 PMC5583076

[12] Ely EW Margolin R Francis J May L Truman B Dittus R Evaluation of delirium in critically ill patients: validation of the confusion assessment method for the intensive care unit (CAM-ICU). Crit Care Med. (2001) 29(7):1370–9. 10.1097/00003246-200107000-00012 11445689

[13] Kim JH Hua M Whittington RA Lee J Liu C Ta CN A machine learning approach to identifying delirium from electronic health records. JAMIA Open. (2022) 5(2):ooac042. 10.1093/jamiaopen/ooac042 35663114 PMC9152701

[14] Bishara A Chiu C Whitlock EL Douglas VC Lee S Butte AJ Postoperative delirium prediction using machine learning models and preoperative electronic health record data. BMC Anesthesiol. (2022) 22(1):8. 10.1186/s12871-021-01543-y 34979919 PMC8722098

[15] Corradi JP Thompson S Mather JF Waszynski CM Dicks RS. Prediction of incident delirium using a random forest classifier. J Med Syst. (2018) 42(12):261. 10.1007/s10916-018-1109-0 30430256

[16] Racine AM Tommet D D’Aquila ML Fong TG Gou Y Tabloski PA Machine learning to develop and internally validate a predictive model for post-operative delirium in a prospective, observational clinical cohort study of older surgical patients. J Gen Intern Med. (2021) 36(2):265–73. 10.1007/s11606-020-06238-7 33078300 PMC7878663

[17] Young M Holmes N Kishore K Marhoon N Amjad S Serpa-Neto A Natural language processing diagnosed behavioral disturbance vs confusion assessment method for the intensive care unit: prevalence, patient characteristics, overlap, and association with treatment and outcome. Intensive Care Med. (2022) 48(5):559–69. 10.1007/s00134-022-06650-z 35322288 PMC9050783

[18] Puelle MR Kosar CM Xu G Schmitt E Jones RN Marcantonio ER The language of delirium: keywords for identifying delirium from medical records. J Gerontol Nurs. (2015) 41(8):34–42. 10.3928/00989134-20150723-01 26248142 PMC4551393

[19] Crema C Attardi G Sartiano D Redolfi A. Natural language processing in clinical neuroscience and psychiatry: a review. Front Psychiatry. (2022) 13:946387. 10.3389/fpsyt.2022.946387 36186874 PMC9515453

[20] Chen PF Chen KC Liao WC Lai F He TL Lin SC Automatic international classification of diseases coding system: deep contextualized language model with rule-based approaches. JMIR Med Inform. (2022) 10(6):e37557. 10.2196/37557 35767353 PMC9282222

[21] Johnson AEW Pollard TJ Shen L Lehman LwH Feng M Ghassemi M MIMIC-III, a freely accessible critical care database. Sci Data. (2016) 3(1):160035. 10.1038/sdata.2016.35 27219127 PMC4878278

[22] Bodenreider O. The unified medical language system (UMLS): integrating biomedical terminology. Nucleic Acids Res. (2004) 32(Database issue):D267–70. 10.1093/nar/gkh061 14681409 PMC308795

[23] Santos A Colaço AR Nielsen AB Niu L Geyer PE Coscia F Clinical knowledge graph integrates proteomics data into clinical decision-making. bioRxiv. (2020); 10.1101/2020.05.09.084897

[24] Kraljevic Z Searle T Shek A Roguski L Noor K Bean D Multi-domain clinical natural language processing with MedCAT: the medical concept annotation toolkit. Artif Intell Med. (2021) 117. 10.1016/j.artmed.2021.102083 34127232

[25] Bendayan R Kraljevic Z Shaari S Das-Munshi J Leipold L Chaturvedi J Mapping multimorbidity in individuals with schizophrenia and bipolar disorders: evidence from the south London and maudsley NHS foundation trust biomedical research centre (SLAM BRC) case register. BMJ Open. (2022) 12(1):e054414. 10.1136/bmjopen-2021-054414 35074819 PMC8788233

[26] Beam AL Kompa B Schmaltz A Fried I Weber G Palmer N Clinical concept embeddings learned from massive sources of multimodal medical data. Pac Symp Biocomput. (2020) 25:295–306. 10.1142/9789811215636_0027 31797605 PMC6922053

[27] McCormick N Lacaille D Bhole V Avina-Zubieta JA. Validity of heart failure diagnoses in administrative databases: a systematic review and meta-analysis. PLoS One. (2014) 9(8):e104519. 10.1371/journal.pone.0104519 25126761 PMC4134216

[28] Walraven CV. A comparison of methods to correct for misclassification bias from administrative database diagnostic codes. Int J Epidemiol. (2018) 47(2):605–16. 10.1093/ije/dyx253 29253160

