# Supplementary material for: Identifying encephalopathy in patients admitted to an intensive care unit: Going beyond structured information using natural language processing
Source: Front Digit Health. 2023 Jan 23;5:1085602. doi: 10.3389/fdgth.2023.1085602 (PMC9899891; doi:10.3389/fdgth.2023.1085602)
Supplement: Supplementary file 1 [file Table1.docx]

**Supplementary Material**

1. Final CCRE-CUIs
2. Performance of MedCAT against expert curation

Final CCRE-CUIs

An initial selection of 29 ICD-9 codes ( '2930', '2931', '29281', '29011', '2903', '29041', '2910','2939', '78009', '29381', '29382', '29383', '29384', '29389', '29012', '29013', '29043', '29211', '29212', '2922', '78002', '2902', '29042', '2908', '2909', '2920', '29282', '3483', '34831', '34839', '34982', '78097') was mapped into a knowledge graph using UMLS ontology in order to expand their correspondent CUIs to its children concepts. This list was partially curated to exclude diagnoses that are not always associated with encephalopathy ("alcohol withdrawal", "concussion" and "psychosis") and include signs or symptoms of encephalopathy that don’t have an ICD-9 code of a disease diagnosis (CUI C0085631 for “Agitation”, CUI C0011206 for “Delirium”, CUI C0023380 for “Lethargy”, CUI C0009676 for “Confusion” and CUI C0237284 for “unresponsive”). This resulted in 1498 different CCRE-CUIs and 40 ICD-9 codes when mapped back through the knowledge graph.

Performance of MedCAT against expert curation

As a validation process of the MedCAT output, we manually annotated 50 random documents with the condition that had the concept “encephalopathy” with the annotation tool of the MedCAT application.

The average accuracy of clinical concepts was 86.7%.

The average accuracy of annotating a negation was 95.9%

The average accuracy of annotating an irrelevant concept is 91.2%
